# Evaluation of Olfactory Dysfunction Among COVID-19 Patients in Baghdad, Iraq

**DOI:** 10.7759/cureus.53721

**Published:** 2024-02-06

**Authors:** Mohammad F Kasim, Azzam M Abbas

**Affiliations:** 1 Otolaryngology, Ninveha Health Directorate, Mosul, IRQ; 2 Otolaryngology, College of Medicine, University of Baghdad and Martyr Ghazi Al-Hariri Teaching Hospital, Baghdad Medical City, Baghdad, IRQ

**Keywords:** sars-cov-2, otolaryngology, iraq, smell test, gustatory dysfunction, hyposmia, anosmia

## Abstract

Background

SARS‑CoV‑2 (COVID-19) causes olfactory dysfunction which is characterized by anosmia or hyposmia. Characterization of olfactory dysfunction has added value to the diagnosis and prognosis of the disease. Nevertheless, scarce information exists about COVID-19 patients suffering from olfactory dysfunction in Iraq. This study aimed to identify olfactory dysfunction (anosmia or hyposmia) in Iraqi COVID-19 patients and examine their response to smell exercise at Baghdad Medical City Complex, Baghdad, Iraq.

Methodology

This case series prospective study involving 300 patients (160 males and 140 females) with COVID-19 infection was conducted from June 1, 2020, to October 1, 2021. We recorded signs and symptoms of COVID-19 among patients by examining olfactory dysfunction, n-butanol olfaction test, and smell test exercise.

Results

Anosmia and hyposmia were found in 69.3% and 30.7% of the patients, respectively; of these, 65.7% were of sudden onset. The association between olfactory dysfunction and smoking was not significant. The most frequent signs and symptoms of COVID-19 were fatigue, fever, loss of taste, myalgia, headache, sore throat, cough, depressed appetite, dyspnea, nausea, abdominal pain, and diarrhea. The highest frequencies of occurrence of anosmia (30.7%) and hyposmia (13.3%) were in the age group of 31-40 years. The majority (47.7%) of patients with olfactory dysfunction recovered within one month of COVID-19 onset. The rest of the patients recovered within one month to 16 months. The most commonly encountered ear, nose, and throat symptoms were nasal obstruction, rhinorrhea, and facial/ear pain. The percentages of patients with anosmia and hyposmia recovering with smell exercise were significant at 64.7% and 25.3%, respectively.

Conclusions

The prognosis of olfactory dysfunction in COVID-19 patients was good as most cases recovered within a short period with concomitant smell exercise. Olfactory dysfunction in the majority of COVID-19 patients was self-limiting in young age groups, albeit in association with the non-severity of the disease. Being an important public health issue, examining olfactory dysfunction aspects should be considered in the diagnosis, prognosis, and treatment protocols of COVID-19 patients. In-depth exploration is needed to examine olfactory and gustatory dysfunction in patients suffering from severe COVID-19.

## Introduction

COVID-19 caused by SARS‑CoV‑2 is a major public health concern characterized by high morbidity and significant loss of lives due to the dysfunction of several organ systems [1,2]. COVID-19 patients with acute respiratory syndrome suffer from respiratory distress due to alveolar damage which might result in progressive pneumonia and respiratory failure [2]. This respiratory affection is mostly accompanied by a deterioration in the smelling ability of the patient who might experience anosmia (absence of smell) or hyposmia (decreased sensitivity to odors) [3,4], which affects the quality of life and safety [5]. Following COVID-19, olfactory and gustatory dysfunctions are important signs that might aid clinicians in managing the disease [3,6]. Human strains of SARS‑CoV‑2 invade the brain through the neuroepithelium up to the olfactory bulb, contributing considerably to anosmia in infected patients [6-8]. However, non-symptomatic patients or those with mild symptoms of COVID-19 might present with anosmia or hyposmia [9,10].

Numerous studies from several countries have reported olfactory and gustatory dysfunctions among COVID-19 patients that can be sudden in onset regardless of the disease outcome [6,10-12]. These dysfunctional aspects of COVID-19 are frequently reported; however, they can be ignored or underrated [13]. It should be stressed that testing for olfactory and gustatory dysfunctions can aid in the diagnosis of COVID-19 [3,4]. Even testing and retesting of patients with negative COVID-19 polymerase chain reaction (PCR) results have been practiced to aid in the diagnosis of the disease [12]. Numerous reports exist on the prevalence of various strains of SARS‑CoV‑2 in different regions of Iraq causing acute clinical manifestations that include the respiratory system and other organ systems [14-19].

Based on the literature cited above, whether in Iraq or elsewhere, anosmia accompanied by ageusia (loss of taste) or bitter taste can be significant findings among COVID-19 patients [5,6,9,11-14]. Within this context, COVID-19 patients might suffer from olfactory and gustatory disturbances in the early stages of the disease during which fewer symptoms or no nasal discharges are apparent [10-13,20]. Therefore, it is essential to characterize olfactory dysfunction in COVID-19 patients with the added value in the diagnosis and evaluation of the prognosis of the disease. Nevertheless, scarce information exists about COVID-19 patients suffering from olfactory dysfunction in Iraq. Therefore, it was pertinent to further identify the condition of anosmia or hyposmia in Iraqi COVID-19 patients due to the importance of olfactory dysfunction in the quality of life and safety [5]. This study aimed to assess anosmia or hyposmia among COVID-19 PCR-positive patients and their response to smell exercise at Baghdad Medical City Complex, Baghdad, Iraq.

## Materials and methods

Ethical approval

This study was approved by the Institutional Review Board of the Iraqi Board for Medical Specialties, Baghdad, Iraq (reference number: 3448). The study was conducted following the guidelines of the Declaration of Helsinki. Written consent was obtained from each patient recruited for the study. We informed the patients about the purpose of the study, methods of data collection, the tests to be conducted, the expected outcomes of the study, and potential recommendations. All patient data and information were kept confidential.

Inclusion criteria

Inclusion criteria comprised COVID-19 patients with mild (e.g., fever, fatigue, cough, without dyspnea) or moderate (clinical or radiographic evidence of lower respiratory tract disease, oxygen saturation <94) illness presentation. All patients subjectively were suffering from a loss of smell that was persistent for more than one week after the onset of COVID-19 symptoms. The study included patients aged >18 years old to ensure the accuracy of information provided during the interview, obtain disease history, and ensure full cooperation during examination and follow-up, as well as treatments and instructions.

Exclusion criteria

Exclusion criteria included patients with severe COVID-19, those uncooperative during data acquisition and clinical examination, those with olfactory and/or gustatory disorders before the COVID-19 pandemic in Iraq, those with a previous history of nasal surgery, and those with a previous history of head injury. Patients with previously diagnosed chronic rhinosinusitis or those with nasal mass or on any medications causing olfactory disturbance were also excluded from the study. Hospitalized patients or those suffering from severe COVID-19 were also excluded from the study.

Patients, study design, and location

This case series prospective study involved 300 patients with COVID-19 (PCR-positive COVID-19) suffering from olfactory dysfunction. The study was conducted at the Department of Ear, Nose, and Throat (ENT), Martyr Ghazi Al-Hariri Hospital for Surgical Specialties, Baghdad Medical City Complex from June 1, 2020, to October 1, 2021. A total of 300 COVID-19 PCR-positive patients of both genders (160 males and 140 females) who met the inclusion criteria were recruited from the outpatient clinic, as well as via referrals by primary healthcare centers.

The information obtained from each patient at the interview included age, gender, occupation, residency, smoking habit, the onset of olfactory dysfunction (anosmia and/or hyposmia), and duration of loss of smell. Recorded signs included unilateral or bilateral nasal obstruction and whether it was transient or persistent, the color of the nasal discharge, the quantity and consistency of the nasal discharge, and whether it was mixed with blood or not, as well as any concurrent occurrence of headache and facial pain. We also recorded the occurrence of any foul smell within the nose, loss of taste, and general signs and symptoms of COVID-19 infection such as fever cough, fatigability, and dyspnea.

The ENT checkup of each patient included the external nasal framework, anterior rhinoscopy, and Hopkin zero-degree rigid nasal endoscopy, along with an examination of tenderness over paranasal sinuses, nasal patency test using cold spatula test and Cottle’s test. Additionally, oropharyngeal and ear examinations were performed as well. We used olfaction test developed at the Connecticut Chemosensory Clinical Research Center, which has an n-butanol threshold component and an odor identification component with a composite score [21,22]. Each side of the nostril was tested separately. Briefly, the strongest concentration of n-butanol (4%) was in 60 mL of deionized water (bottle zero), and other bottles (one to eight) contained 1:3 n‐butanol dilutions. The bottle was brought within 2 cm of the nostrils and allowed to be sniffed for three to four seconds, and we recorded the bottle that produced the smell most. The threshold was identified with four correct answers. In case of an error, the next most concentrated solution was given. Furthermore, in the identification test of odors of edible stimuli [23], we used five common items (coffee, lemon, garlic, mint, and banana). Samples, one at a time, in opaque jars were presented to each patient to be sniffed and to identify the odor by holding the irrelevant nostril closed.

Smell exercise

Each patient was instructed to perform a self-smell exercise [24] using five different materials that included lemon, clove, mint, vanilla, ground pepper, and garlic, with the usage of ground coffee as a neutralizing material. Each material was placed in a separate small jar. The patient was instructed to relax and take short gentle sniffs (not too deep, nor quick) for 20 seconds. The patient was then instructed to repeat the training two to three times for each material and take a five-minute break between materials. The training schedule was repeated twice a day. Patient follow-up was done by instructing them to regularly visit the outpatient clinic, or by telephone every two weeks until recovery from olfactory dysfunction. The patients had no specific antiviral pharmacological treatments. However, they were given alpha-lipoic acid 600 mg/day, omega III 1 g twice daily, with a daily 8 mg zinc supplement until reaching recovery.

Statistical analysis

Descriptive univariate statistics were applied to the data using the statistical package PAST4.14 (https://www.nhm.uio.no/english/research/resources/past/). Frequency data were analyzed by the chi-square test, and non-parametric data were analyzed by the Mann-Whitney U test at a two-tailed probability test. Statistical significance was set at p-values <0.05.

## Results

As shown in Table 1, this study included 300 COVID-19 PCR-positive patients with olfactory dysfunction. Their mean age ± SD was 34.3 ± 8.3 years. Overall, 53.3% were males and 46.7% were females. Anosmia and hyposmia were found in 69.3% and 30.7% of the patients, respectively. Of these, 65.7% were of sudden onset, and 34.3% were of gradual onset. Moreover, 2.7% of the patients required admission to the emergency causality unit for supportive treatment due to COVID-19 manifestations. The majority of patients who developed anosmia (81.7%) and hyposmia (80.4%) were non-smokers, and the association between olfactory dysfunction and smoking was not significant (χ^2^ = 0.07, df = 1, p = 0.79) (Table 2).

**Table 1:** Characteristics of COVID-19 patients suffering from olfactory dysfunction. SD: standard deviation; COVID-19: coronavirus disease 2019

Characteristics	Number	%	Minimum-maximum (year)	Mean ± SD (year)
Total patients	300	100	21–72	34.3 ± 8.3
Males	160	53.3	21–72	34.9 ± 8.7
Females	140	46.7	21–61	33.6 ± 7.8
Olfactory dysfunction				
Anosmia	208	69.3	-	-
Hyposmia	92	30.7	-	-
Onset pattern				
Sudden onset	197	65.7	-	-
Gradual onset	103	34.3	-	-
Admitted to emergency causality	8	2.7	-	-

**Table 2:** Association of smoking with olfactory dysfunction in COVID-19 patients. *: The association was not significant (χ^2^ = 0.07, df = 1, p = 0.79). COVID-19: coronavirus disease 2019

Characteristics	Anosmia*	%	Hyposmia	%
Non-smoker	170	81.7	74	80.4
Smoker	38	18.3	18	19.6
Total	208	100	92	100

The signs and symptoms of COVID-19 observed in the patients are presented in Table 3. Fatigue was the most common symptom (78.3%) encountered in the patients, followed by fever (70%), loss of taste (68.7%), myalgia (57.7%), headache (53.3%), sore throat (53%), cough (45%), depressed appetite (43.3%), dyspnea (21.7%), nausea (17.3%), abdominal pain (13%), and diarrhea (7.3%), whereas palpitation, chest pain, and insomnia were encountered at 0.3% to 0.7%. The highest frequency of occurrence of olfactory dysfunction, anosmia, and hyposmia was in the age group of 31-40 years at 30.7% and 13.3%, respectively (Table 4). However, the occurrence of olfactory dysfunction gradually declined in older age groups at 0.7% for anosmia and hyposmia in the age group >60 years. As shown in Table 5, the most common ENT symptoms encountered among COVID-19 patients were nasal obstruction (45.7%), followed by rhinorrhea (36%), and facial/ear pain (33%), whereas epistaxis was found to be the least symptom encountered in 2.3% of the patients.

**Table 3:** General symptoms associated with COVID-19 in 300 patients with anosmia and hyposmia. COVID-19: coronavirus disease 2019

Symptoms	Number of patients	%
Fatigue	235	78.3
Fever	210	70.0
Loss of taste	206	68.7
Myalgia	173	57.7
Headache	160	53.3
Sore throat	159	53.0
Cough	135	45.0
Decreased appetite	130	43.3
Dyspnea	65	21.7
Nausea	52	17.3
Abdominal pain	39	13.0
Diarrhea	22	7.3
Palpitation	2	0.7
Chest pain	1	0.3
Insomnia	1	0.3

**Table 4:** Occurrence of anosmia and hyposmia in 300 COVID-19 patients according to age groups. COVID-19: coronavirus disease 2019

Age groups (years)	Anosmia	%	Hyposmia	%
20–30	84	28.0	36	12.0
31–40	92	30.7	40	13.3
41–50	27	9.0	12	4.0
51–60	3	1.0	2	0.7
>60	2	0.7	2	0.7
Total	208	69.3	92	30.7

**Table 5:** ENT-related symptoms of 300 COVID-19 patients with olfactory dysfunction. ENT: ear, nose, and throat; COVID-19: coronavirus disease 2019

Complaints	Number of patients	%
Nasal obstruction	137	45.7
Rhinorrhea	108	36.0
Facial/Ear pain	99	33.0
Sneezing	81	27.0
Nasal burning sensation	70	23.3
Post-nasal discharge	50	16.7
Throat congestion	40	13.3
Epistaxis	7	2.3

The majority (47.7%) of patients with olfactory dysfunction (anosmia and hyposmia) recovered within one month of COVID-19 onset (Table 6). The second largest group represented 25% of the study population in which the duration of recovery ranged between 1-2 months, whereas the longest recovery period was 10-16 months noticed in 1.7% of the patients. The percentage of patients who recovered from olfactory dysfunction within the observation period of 16 months was 90% (Table 6). Thereafter, 30 (10%) patients unfortunately dropped out of the follow-up period.

**Table 6:** Duration of olfactory dysfunction (anosmia and hyposmia) in 300 COVID-19 patients until recovery. A total of 30 (10%) patients could not be reached after 16 months of follow-up. COVID-19: coronavirus disease 2019

Duration (month)	Number of patients	%
Up to 1	143	47.7
1.1–2	75	25.0
2.1–3	17	5.7
3.1–4	11	3.7
4.1–5	2	0.7
5.1–6	6	2.0
6.1–7	5	1.7
7.1–8	2	0.7
8.1–9	2	0.7
9.1–10	2	0.7
10.1–16	5	1.7
Total	270	90.0

The percentages of COVID-19 patients with anosmia and hyposmia recovering with the use of smell exercise were 64.7% and 25.3%, respectively, compared to 4.7% and 5.3% of non-recovered patients, respectively, even with the usage of smell exercise (Table 7). Accordingly, the association of smell exercise with the recovery from anosmia or hyposmia was statistically significant as determined (χ^2^ = 8.0546, df = 1, and p < 0.0045). Comparison of recovery periods (in months) of anosmia (1.61 ± 2.01) and hyposmia (1.88 ± 2.29) with their medians of one month each indicated no significant statistical difference (Table 8).

**Table 7:** Relationship between recovery of COVID-19 patients from olfactory dysfunction and the use of smell exercise during 1-16 months. The association was significant (χ^2^ = 8.0546, df = 1, p < 0.0045). COVID-19: coronavirus disease 2019

Olfactory dysfunction	Affected	Recovered with smell exercise	%	Unrecovered with smell exercise	%
Anosmia	208	194	64.7	14	4.7
Hyposmia	92	76	25.3	16	5.3
Total	300	270	90.0	30	10

**Table 8:** Recovery periods of COVID-19 patients from anosmia and hyposmia. The recovery times of anosmia and hyposmia were not significantly different (Mann-Whitney U test, two-tailed probability, p = 0.611). COVID-19: coronavirus disease 2019

Statistics	Anosmia	Hyposmia
Number	208	92
Mean recovery time (month)	1.61	1.88
Standard deviation	2.01	2.29
Standard error	0.12	0.22
Minimum recovery time (month)	0.25	0.25
Maximum recovery time (month)	16	10
95% confidence interval for the mean	1.38, 1.85	1.45, 2.31
Median recovery time (month)	1	1

## Discussion

Loss of smell remarkably affects the quality of life and safety of patients suffering from viral diseases, including COVID-19 [5]. Anosmia and hyposmia are prominent signs of olfactory dysfunction in SARS-CoV-2 infection [3,4,6]. However, olfactory dysfunction might occur suddenly in COVID-19 patients without any other apparent severe complication of the disease [4-6]. In this study, we characterized the outcome of 300 COVID-19 patients suffering from anosmia (69.3%) and hyposmia (30.7%). While the COVID-19 symptoms were not life-threatening, generalized inflammatory responses were encountered among the patients. These included fatigue, fever, loss of taste, myalgia, headache, sore throat, coughing, depressed appetite, dyspnea, nausea, bitter taste and/or loss of taste, abdominal pain, and diarrhea. These conditions also coincided with the fact that only 2.7% of patients with olfactory dysfunction required admission to the causality unit for further supportive treatment, and none of the patients died. However, the related ENT symptoms among the patients in this study support the impact of the disease on the naso-oro-pharyngo-laryngeal airway, but with no clear relationships with olfactory and gustatory dysfunctions. This is probably because the conditions of COVID-19 patients were not severe enough, as indicated in the selection criteria of the study. Therefore, we recommend addressing olfactory dysfunction in association with the severity of ENT symptoms in future studies. In addition, olfactory dysfunction in COVID-19 patients should be frequently monitored as this would aid in monitoring and predicting the condition of the patient, especially in the early stages of the disease, as olfactory dysfunction can be a risk factor when the ENT symptoms persist for long periods [5,6,9,10-13,20].

In this study, young (20-30 years) and younger adults (31-40 years) among other age groups (41-60 and more) were the most susceptible to anosmia (28%-30.7%) and hyposmia (12%-13.3%). This finding of younger age susceptibility to olfactory dysfunction correlates with other reports on COVID-19 patients [25,26]. However, olfactory dysfunction can occur in COVID-19 patients irrespective of gustatory dysfunction [27]. Nevertheless, older COVID-19 patients were also reported to suffer more from olfactory dysfunction [28]. It should be stressed that as humans become older the ability to discriminate among different smells declines and the sensitivity to the smell sensation decreases [29]. The pathophysiology of COVID-19-induced olfactory dysfunction can be attributed to disruptions of peripheral (nasal cavity) and central (olfactory centers) neuronal mechanisms involved in the processes of olfaction [25,30,31]. Nasal obstruction with underlying inflammatory edema can be an early cause of olfactory dysfunction in COVID-19 patients [31]. In this study, nasal obstruction occurred in 45.7% of the patients with other major ENT symptoms that ranged from 16.7% to 36% (Table 5).

In this study, 160 (53.3%) patients with olfactory dysfunction were males, with a slight non-significant increase over females (46.7%). This result is in agreement with those of others [32-34] but contradicts other studies in which the percentage of olfactory dysfunction in females (63.1%) was higher than that in males (36.9%) [35]. COVID-19 olfactory dysfunction due to gender differences is not yet clear; however, it can be related to the inherent gender differences in response to infectious inflammatory response [36].

The high percentage (65.7%) of sudden onset of olfactory dysfunction compared to that of the gradual one (34.3%) is consistent with other studies conducted among COVID-19 patients [25,26]. The sudden onset of olfactory dysfunction has been suggested to be an important outcome of acute COVID-19 infection, and it is considered of utmost public health concern that can be used as an early predictor for COVID-19 infection [13,35,37-39]. In this study, loss of taste was associated with almost all cases of anosmia, accounting for 68.8% of the total COVID-19 patients enrolled in the study. Gustatory dysfunction is a common finding with olfactory dysfunction in COVID-19 patients [10,32-35]. It has been argued that gustatory dysfunction can occur independently from olfactory dysfunction [27]. To this end, it has been suggested that mild clinical signs and symptoms of COVID-19, as reported in the present study, together with olfactory and gustatory dysfunctions can have a strong association with the clinical course of COVID-19 [39]. This, in turn, would aid in taking certain precautionary measures in COVID-19 isolation and considering PCR diagnostic testing and commencement of therapy as soon as possible.

Smoking among the COVID-19 patients of the present study was not associated with the occurrence of olfactory dysfunction. Although similar findings have been reported earlier among COVID-19 patients, olfactory affection with or without smoking and disease progression remain uncertain [40,41].

As reported in other studies [26,35,42], the majority of the COVID-19 patients who suffered from olfactory dysfunction in this study recovered within two months; the mean and median of the recovery period were 1.6 and 1.8 months, respectively. Early recovery of olfactory dysfunction, however, is not always an expected outcome among COVID-19 patients [35], which should be taken into consideration clinically [43]. We observed delayed recovery (beyond six months) in about 5% of our patients.

The association of smell exercise with recovery from anosmia or hyposmia was statistically significant among the COVID-19 patients of this study. The percentages of patients recovering from anosmia and hyposmia with the use of smell exercise were 64.7% and 25.3%, respectively. This finding, which is similar to those of others [43,44], appears to be of clinical value, as the practice of smell exercise, being associated with minimal harmful effects, could benefit the patient by improving the olfactory function and discrimination. We recommend commencing smell exercises as early as possible with strict high compliance. The only drawback appears to be the need for sustained daily training for a long period that could extend to months. It is also possible that daily supplements of the patients with alpha-lipoic acid, omega III, and zinc helped them to recover from olfactory dysfunction. However, no specific antiviral pharmacological treatments were given to the patients. Olfactory dysfunction caused by COVID-19 can persist in some patients; however, the infection may not permanently disrupt the olfactory epithelium in most cases and leave a subgroup of olfactory neurons intact for normal physiological functioning [42-45].

Limitations of the study

The study was selective and did not target all COVID-19 patients regardless of the severity of the illness and the occurrence of olfactory dysfunction. The study did not consider the association of olfactory dysfunction with different variants of SARS-CoV-2. A longer follow-up period could have delineated the outcome of 30 (10%) COVID-19 patients in the present study. However, they dropped out of contact after 16 months of follow-up, and we were unable to verify their recovery from olfactory dysfunction. Despite the limitations, the results of this study emphasize the value of considering olfactory dysfunction in the assessment of the prognosis of COVID-19 with longer follow-ups to investigate various functional aspects of olfactory and gustatory dysfunctions.

## Conclusions

The prognosis of olfactory dysfunction in COVID-19 patients was good, as recovery in most cases occurred in a short period with concomitant smell exercises. Olfactory dysfunction in a majority of COVID-19 patients was self-limiting in young age groups, albeit in association with the non-severity of the disease. Being an important public health issue, examining olfactory dysfunction should be considered in the diagnosis, prognosis, and treatment protocols of COVID-19 patients. However, in-depth exploration is needed to examine olfactory and gustatory dysfunctions in patients suffering from severe COVID-19 and/or those with prolonged anosmia to identify vital management and rehabilitation approaches.

## References

[1] Vosko I, Zirlik A, Bugger H (2023). Impact of COVID-19 on cardiovascular disease. Viruses.

[2] Jeong YJ, Wi YM, Park H, Lee JE, Kim SH, Lee KS (2023). Current and emerging knowledge in COVID-19. Radiology.

[3] Alkholaiwi FM, Altamimi AF, Almalki HH (2023). Olfactory dysfunction among patients with COVID-19. Saudi Med J.

[4] Aziz M, Goyal H, Haghbin H, Lee-Smith WM, Gajendran M, Perisetti A (2021). The association of "loss of smell" to COVID-19: a systematic review and meta-analysis. Am J Med Sci.

[5] Coelho DH, Reiter ER, Budd SG, Shin Y, Kons ZA, Costanzo RM (2021). Quality of life and safety impact of COVID-19 associated smell and taste disturbances. Am J Otolaryngol.

[6] Tong JY, Wong A, Zhu D, Fastenberg JH, Tham T (2020). The prevalence of olfactory and gustatory dysfunction in COVID-19 patients: a systematic review and meta-analysis. Otolaryngol Head Neck Surg.

[7] Reza-Zaldívar EE, Hernández-Sapiéns MA, Minjarez B (2020). Infection mechanism of SARS-COV-2 and its implication on the nervous system. Front Immunol.

[8] Lukiw WJ, Pogue A, Hill JM (2022). SARS-CoV-2 infectivity and neurological targets in the brain. Cell Mol Neurobiol.

[9] Taziki Balajelini MH, Vakili MA, Saeidi M, Tabarraei A, Hosseini SM (2020). Using anti-SARS-CoV-2 IgG and IgM antibodies to detect outpatient cases with olfactory and taste disorders suspected as mild form of COVID-19: a retrospective survey. SN Compr Clin Med.

[10] Vaira LA, Hopkins C, Salzano G (2020). Olfactory and gustatory function impairment in COVID-19 patients: Italian objective multicenter-study. Head Neck.

[11] Mehraeen E, Behnezhad F, Salehi MA, Noori T, Harandi H, SeyedAlinaghi S (2021). Olfactory and gustatory dysfunctions due to the coronavirus disease (COVID-19): a review of current evidence. Eur Arch Otorhinolaryngol.

[12] Klopfenstein T, Zahra H, Kadiane-Oussou NJ (2020). New loss of smell and taste: uncommon symptoms in COVID-19 patients on Nord Franche-Comte cluster, France. Int J Infect Dis.

[13] Carignan A, Valiquette L, Grenier C (2020). Anosmia and dysgeusia associated with SARS-CoV-2 infection: an age-matched case-control study. CMAJ.

[14] Raouf GA, Mohammad FK, Merza MA (2023). Polypharmacy and the in silico prediction of potential body proteins targeted by these drugs among hospitalized COVID-19 patients with cytokine storm. Cureus.

[15] Raouf GAM, Qader MK, Abdullah IM, Younis OM, Mohammad FK, Merza MA (2023). Detection of SARS-CoV-2 variants among travelers crossing the northern international border checkpoint in Duhok province, Iraq. J Ideas Health.

[16] Merza MA, Almufty HB (2022). The Omicron variant is striking Iraqi Kurdistan in January 2022: would preventive measures contain the new wave?. Disaster Med Public Health Prep.

[17] Merza MA, Haleem Al Mezori AA, Mohammed HM, Abdulah DM (2020). COVID-19 outbreak in Iraqi Kurdistan: the first report characterizing epidemiological, clinical, laboratory, and radiological findings of the disease. Diabetes Metab Syndr.

[18] Al-Rashedi NA, Alburkat H, Hadi AO (2022). High prevalence of an alpha variant lineage with a premature stop codon in ORF7a in Iraq, winter 2020-2021. PLoS One.

[19] Hussein NR, Rashad BH, Almizori LA (2021). The risk of SARS-CoV-2 reinfection in Duhok city, Kurdistan region of Iraq. Mediterr J Hematol Infect Dis.

[20] Altundag A, Yıldırım D, Tekcan Sanli DE (2021). Olfactory cleft measurements and COVID-19-related anosmia. Otolaryngol Head Neck Surg.

[21] Cain WS, Gent JF, Goodspeed RB, Leonard G (1988). Evaluation of olfactory dysfunction in the Connecticut Chemosensory Clinical Research Center. Laryngoscope.

[22] Jegatheeswaran L, Gokani SA, Luke L (2023). Assessment of COVID-19-related olfactory dysfunction and its association with psychological, neuropsychiatric, and cognitive symptoms. Front Neurosci.

[23] Fusari A, Ballesteros S (2008). Identification of odors of edible and nonedible stimuli as affected by age and gender. Behav Res Methods.

[24] Rimmer A (2021). Sixty seconds on . . . smell training. BMJ.

[25] Bagheri SH, Asghari A, Farhadi M (2020). Coincidence of COVID-19 epidemic and olfactory dysfunction outbreak in Iran. Med J Islam Repub Iran.

[26] Amer MA, Elsherif HS, Abdel-Hamid AS, Elzayat S (2020). Early recovery patterns of olfactory disorders in COVID-19 patients; a clinical cohort study. Am J Otolaryngol.

[27] Nguyen H, Albayay J, Höchenberger R (2023). Covid-19 affects taste independently of smell: results from a combined chemosensory home test and online survey from a global cohort (N=10,953). medRxiv.

[28] Liu N, Yang D, Zhang T, Sun J, Fu J, Li H (2022). Systematic review and meta-analysis of olfactory and gustatory dysfunction in COVID-19. Int J Infect Dis.

[29] Hoffman HJ, Rawal S, Li CM, Duffy VB (2016). New chemosensory component in the U.S. National Health and Nutrition Examination Survey (NHANES): first-year results for measured olfactory dysfunction. Rev Endocr Metab Disord.

[30] von Bartheld CS, Hagen MM, Butowt R (2020). Prevalence of chemosensory dysfunction in COVID-19 patients: a systematic review and meta-analysis reveals significant ethnic differences. ACS Chem Neurosci.

[31] Hu B, Gong M, Xiang Y, Qu S, Zhu H, Ye D (2023). Mechanism and treatment of olfactory dysfunction caused by coronavirus disease 2019. J Transl Med.

[32] Al-Ani RM, Acharya D (2022). Prevalence of anosmia and ageusia in patients with COVID-19 at a primary health center, Doha, Qatar. Indian J Otolaryngol Head Neck Surg.

[33] Galluzzi F, Rossi V, Bosetti C, Garavello W (2021). Risk factors for olfactory and gustatory dysfunctions in patients with SARS-CoV-2 infection. Neuroepidemiology.

[34] Gupta V, Banavara Rajanna L, Upadhyay K, Bhatia R, Madhav Reddy N, Malik D, Srivastava A (2021). Olfactory and gustatory dysfunction in COVID-19 patients from northern India: a cross-sectional observational study. Indian J Otolaryngol Head Neck Surg.

[35] Lechien JR, Chiesa-Estomba CM, De Siati DR (2020). Olfactory and gustatory dysfunctions as a clinical presentation of mild-to-moderate forms of the coronavirus disease (COVID-19): a multicenter European study. Eur Arch Otorhinolaryngol.

[36] Lefèvre N, Corazza F, Valsamis J, Delbaere A, De Maertelaer V, Duchateau J, Casimir G (2019). The number of X chromosomes influences inflammatory cytokine production following toll-like receptor stimulation. Front Immunol.

[37] Kay LM (2022). COVID-19 and olfactory dysfunction: a looming wave of dementia?. J Neurophysiol.

[38] Sedaghat AR, Gengler I, Speth MM (2020). Olfactory dysfunction: a highly prevalent symptom of COVID-19 with public health significance. Otolaryngol Head Neck Surg.

[39] Kanjanaumporn J, Aeumjaturapat S, Snidvongs K, Seresirikachorn K, Chusakul S (2020). Smell and taste dysfunction in patients with SARS-CoV-2 infection: a review of epidemiology, pathogenesis, prognosis, and treatment options. Asian Pac J Allergy Immunol.

[40] Gallus S, Scala M, Possenti I (2023). The role of smoking in COVID-19 progression: a comprehensive meta-analysis. Eur Respir Rev.

[41] Cheng MY, Hsih WH, Ho MW (2021). Younger adults with mild-to-moderate COVID-19 exhibited more prevalent olfactory dysfunction in Taiwan. J Microbiol Immunol Infect.

[42] Riestra-Ayora J, Yanes-Diaz J, Esteban-Sanchez J, Vaduva C, Molina-Quiros C, Larran-Jimenez A, Martin-Sanz E (2021). Long-term follow-up of olfactory and gustatory dysfunction in COVID-19: 6 months case-control study of health workers. Eur Arch Otorhinolaryngol.

[43] Prem B, Liu DT, Besser G (2022). Long-lasting olfactory dysfunction in COVID-19 patients. Eur Arch Otorhinolaryngol.

[44] Hopkins C, Surda P, Whitehead E, Kumar BN (2020). Early recovery following new onset anosmia during the COVID-19 pandemic - an observational cohort study. J Otolaryngol Head Neck Surg.

[45] Niklassen AS, Draf J, Huart C (2021). COVID-19: recovery from chemosensory dysfunction. A multicentre study on smell and taste. Laryngoscope.

